# Comparison of false positive rates for screening breast magnetic resonance imaging (MRI) in high risk women performed on stacked versus alternating schedules

**DOI:** 10.1186/s40064-015-0793-1

**Published:** 2015-02-13

**Authors:** Edress Othman, Jue Wang, Brian L Sprague, Tiffany Rounds, YongLi Ji, Sally D Herschorn, Marie E Wood

**Affiliations:** Department of Medicine, University of Vermont, Burlington, VT USA; Division of Biostatistics, University of Texas Health Science Center, Austin, TX USA; Department of Surgery, Vermont Cancer Center, University of Vermont, Burlington, VT USA; Department of Radiology, University of Vermont, Burlington, VT USA

**Keywords:** Breast cancer screening, Screening breast MRI, False positive, Screening schedule, High risk

## Abstract

**Purpose:**

Breast MRI added to mammography increases screening sensitivity for high-risk women but false-positive (FP) rates are higher and the optimal screening schedule for coordination with mammography is unclear. We compare rates of FP MRI when studies were performed on two different schedules.

**Patients and methods:**

High-risk women at the University of Vermont who had at least 1 MRI and 1 mammogram performed within one year between 2004–2012 were eligible for inclusion in this study. Screening was considered stacked if both studies were performed within 90 days and alternating if studies were 4–8 months apart. False positive was defined in one of three ways.

**Results:**

137 women had screening which met inclusion criteria and 371 MRIs were reviewed. The FP rates were similar for the two schedules when considering BI-RAD 4, 5, 0 or biopsy as a positive test. FP rates were significantly higher for the stacked schedule (18.2 vs. 10.2%, p = 0.026) when considering BI-RADS 3-4-5-0 as positive test, due to the elevated rate of BI-RADS 3 assessments among stacked exams.

**Conclusion:**

False positive rates differ based on the type of exam (baseline or subsequent) and definition of positive but do not differ based on imaging schedule (stacked or alternating); suggesting that women and their providers may choose the imaging schedule they prefer. This is significant as a randomized clinical trial comparing the two schedules is not likely to be performed, given the high cost and large number of women needed for such a study.

## Introduction

The implementation of screening mammography was a major step in the fight against breast cancer, but several studies have demonstrated lower sensitivity of mammography in younger women (Kerlikowske et al. 1993) (Hendrick et al. 1997) and women at increased risk (Kuhl et al. 2005) (Berg et al. 2008). Adding screening breast MRI to mammography for women at increased risk for breast cancer (due to a family history) results in increased sensitivity and decreased interval cancer rates (Brekelmans et al. 2001) (Kriege et al. 2001) (Tilanus-Linthorst et al. 2000) (Kriege et al. 2004) (Weinstein et al. 2009) (Klijn 2010) (Kuhl et al. 2005) (Warner et al. 2004) (Leach et al. 2005). However, the specificity of screening breast MRI is low (79-89%) especially for premenopausal women (Brekelmans et al. 2001) (Kriege et al. 2001) (Tilanus-Linthorst et al. 2000) (Kriege et al. 2004) (Weinstein et al. 2009). This means a high false positive rate which results in additional imaging, biopsy and anxiety in this population (Brekelmans et al. 2001) (Kriege et al. 2001) (Tilanus-Linthorst et al. 2000) (Kriege et al. 2004) (Weinstein et al. 2009).

In 2007, the American Cancer Society (ACS) published recommendations for screening breast MRI in addition to mammography for high risk women which include; all women with a lifetime risk of more than 20% to 25% based on family history, women with BRCA mutation, first-degree untested relative of BRCA carrier, women with Li-Fraumeni syndrome and first-degree relatives, women with Cowden syndrome and first-degree untested relatives, and women who had radiation to chest wall between ages 10 and 30 (Saslow et al. 2007).

The optimum timing of screening studies (should they be done at the same time as screening mammography [“stacked”] or alternating with one study every six months) has not been addressed. The above mentioned studies demonstrating the increased sensitivity when MRI is added to mammogram specified that imaging studies were done within a short period of time (on the same day or less than 90 days apart). There have been no studies comparing the two screening schedules directly. Identifying the optimal screening schedule may reduce the false positive rates and reduce unnecessary biopsies and imaging. The current study was undertaken to compare the false positive rates for screening breast MRI when imaging is done at the same time each year (a stacked schedule) compared to an every six month imaging (an alternating) schedule.

## Patients and methods

Women enrolled in an Institutional Review Board (IRB) approved prospective study of women at moderate and high risk for breast cancer between 2004 and 2012 at the University of Vermont were the subject of this current study. To be eligible to participate in the IRB approved database participants must have an increased risk for developing breast cancer. Patients are identified as having an increased risk for developing breast cancer if they meet any one of the following criteria: A strong family history of breast (male or female) and/or ovarian cancer. Strong family history is defined as one of the following: **a**. Two or more first-degree relatives with breast cancer or ovarian cancer. **b**. One first-degree relative and two or more second- or third degree relatives with breast cancer. **c**. One first-degree relative with breast cancer or ovarian cancer before the age of 50 years. **d**. One first-degree relative with breast cancer and one or more relatives with ovarian cancer. **e**. Two second- or third-degree relatives with breast cancer and one or more with ovarian cancer. **f**. One second- or third-degree relative with breast cancer and two or more with ovarian cancer. **g**. Three or more second- or third-degree relatives with breast cancer. **h**. One first-degree relative with bilateral breast cancer. Individuals with a known genetic abnormality of a breast cancer causing gene in themselves or a family member. Individuals with a prior breast biopsy showing atypical ductal hyperplasia or lobular neoplasia (atypical lobular hyperplasia or lobular carcinoma in-situ). Individuals with a Gail Model breast cancer risk of greater than or equal to 1.66% over the next 5 years or greater than 20% lifetime risk.

To be included in the current study women must have had at least 1 screening breast MRI and 1 mammogram performed within 1 year. Women were excluded if they had a personal history of invasive breast cancer, had a diagnostic screening breast MRI (i.e., an MRI done to evaluate suspicious lesions on mammography or clinical breast examination, or MRI done as part of work-up for breast cancer) or were using chemoprevention. Medical records were abstracted for clinical, radiological, and biopsy data. One cycle of surveillance was defined as 1 MRI and 1 mammogram done within 1 year. Screening was considered stacked if both studies were performed within 90 days and alternating if studies were 4–8 months apart. Three definitions of a false-positive MRI were considered: 1) MRI result of BI-RADS 4, 5, 0 with no cancer diagnosis within 365 days; 2) BI-RADS 3, 4, 5, 0 with no cancer diagnosis within 365 days; and 3) benign breast biopsy after an MRI-based recommendation.

Dynamic contrast enhanced MRI was performed according to standard techniques (which varied over the time course of the study) and interpreted by breast imaging radiologists with 1–12 years’ experience interpreting breast MRI. The original clinical reports were used in this study. The images were not re-analyzed for research purposes.

### Statistics

The analyses were restricted to the 371 MRIs among 137 women which met the above inclusion criteria. Chi-square tests and Fisher’s exact tests were used to compare differences in assessments and false-positive rates between stacked and alternating MRI screens. All statistical analyses were performed using SAS Statistical Software (Version 9.2; SAS Institute, Inc., Cary, North Carolina).

## Results

**Figure 1:**
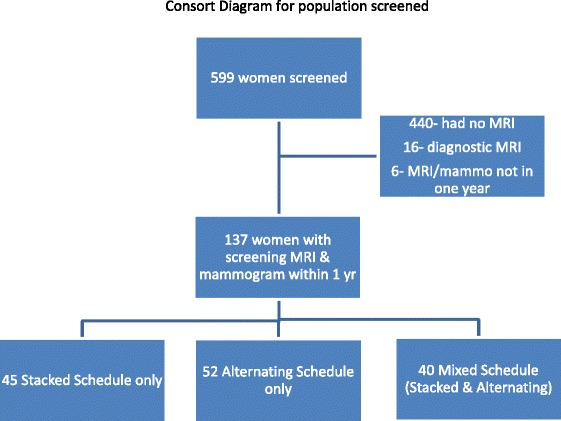
**Consort diagram for population screened.**

The records of 599 women at increased risk of developing breast cancer and enrolled in the parent database at the University of Vermont from May 1^st^, 2004 to March 31^st^, 2012 were reviewed. 440 women did not have a breast MRI as part of their screening, 16 women who had breast MRI performed to evaluate mammographic findings were excluded; an additional 6 women were excluded because they did not have an MRI and mammogram performed within one year. Therefore, 137 women met the inclusion criteria and are the subject of this analysis. See Figure 1 for a consort diagram of study participants. The mean age of women at the time of initial screening breast MRI was 45.8; 78.8% were premenopausal. High risk was attributed to strong family history of breast cancer in 87.6%, BRCA mutation in 18.3%, and prior atypical biopsy in 13.1%. The median number of surveillance cycles was 2 (range of 1 to 7). Table 1 outlines the characteristics of the participants.

**Table 1:** **Baseline characteristics for women with screening breast MRI**

**Characteristic**	**N (%)**
***Demographics***	137
**Age (mean/range)**	45.8 (27 – 77)
**White**	135 (98.5%)
**Premenopausal**	108 (78.8%)
**Postmenopausal**	29 (21.2%)
Reason for screening*	
**Family history**	120 (87.6%)
**BRCA positive**	25 (18.2%)
**Biopsy with atypia or lobular neoplasia**	18 (13.1%)
**Prior chest irradiation**	2 (1.5%)

*Note that individual women could have multiple reasons for screening.

Forty-five women had all screening cycles done on a stacked schedule, 52 women had all cycles done on an alternating schedule, while 40 women had cycles that were mixed with some on a stacked schedule and others on an alternating schedule. 371 screening breast MRIs were reviewed; 165 performed on a stacked and 206 on an alternating schedule. Table 2 reveals the BI-RADS interpretation for MRIs performed on each schedule. There were significantly more BI-RADS category 3 interpretations for MRIs performed on a stacked schedule (9.7 vs. 2.9%, p = 0.006). The likelihood of BI-RADS category 4, 5, or 0 interpretation was not significantly different between the two groups (9.7 vs. 8.3%, p = 0.627). In addition there were fewer BI-RADS category 1 or 2 interpretations for MRIs performed on a stacked schedule (80.6 vs 88.8%, p = 0.027).

**Table 2:** **BI-RADS interpretation according to screening schedule**

**Interpretation**	**Stacked (n = 165)**	**Alternating (n = 206)**	**P value**
	**N (%) [95% CI]**	**N (%) [95% CI]**	
**BI-RADS 1 or 2**	133 (80.6%)	183 (88.8%)	0.027
	[73.9, 86.0]	[83.8, 92.5]	
**BI-RADS 3**	16 (9.7%)	6 (2.9%)	0.006
	[6.0, 15.3]	[1.2, 6.4]	
**BI-RADS 4, 5, 0**	16 (9.7%)	17 (8.3%)	0.627
	[6.0, 15.3]	[5.1, 12.9]	

The false positive rate was first calculated for MRIs with BI-RADS 4,5,0 interpretations demonstrating a non-significant difference between screening on a stacked or alternating schedule (8.5 vs 7.3% respectively, p = 0.668) (Table 3). Defining the false-positive rate for MRIs as BI-RADS 3, 4, 5, 0 interpretation revealed a significant difference between the two screening schedules with 18.2% of MRIs performed on the stacked schedule having a false-positive study compared to 10.2% of MRIs performed on the alternating schedule (p = 0.026). Taking into account weather an MRI was baseline or a subsequent exam eliminated the statistical significance. However stacked exams (both baseline and subsequent) did have a higher rate of false-positive BI-RADS 3, 4, 5, 0 exams compared to alternating exams.

**Table 3:** **False positive rates according to schedule**

	**Overall**	**Stacked**	**Alternating**	**p value**
	**(n = 371)**	**(n = 165)**	**(n = 206)**	
BI-RADS 3*	22 (5.9%)	16 (9.7%)	6 (2.9%)	0.006
	[3.9, 8.9]	[6.0, 15.3]	[1.2, 6.4]	
**BI-RADS 4, 5, 0**	29 (7.8%)	14 (8.5%)	15 (7.3%)	0.668
	[5.5, 11.0]	[5.0, 13.8]	[4.4, 11.8]	
**BI-RADS 3, 4, 5, 0**	51 (13.7%)	30 (18.2%)	21 (10.2%)	0.026
	[10.6, 17.6]	[13.0, 24.8]	[6.7, 15.1]	
**BI-RADS 3, 4, 5, 0**	31 (24.6%)	20 (27.4%)	11 (20.8%)	0.393
**For baseline MRI**	[17.9, 32.8]	[18.4, 38.6]	[11.8, 33.6]	
**BI-RADS 3, 4, 5, 0**	20 (8.2%)	10 (10.9%)	10 (6.5%)	0.230
**For subsequent MRI**	[5.3, 12.3]	[5.8, 19.0]	[3.5, 11.8]	
**Benign biopsy**	22 (5.9%)	10 (6.1%)	12 (5.8%)	0.92
	[3.9, 8.9]	[3.2, 10.9]	[3.3, 10.0]	

*p value compares stacked to alternating schedule.

**Numbers in parentheses are percentages, and numbers in square brackets are the 95% Agresti-Coull confidence intervals for the percentage.

We then considered false-positive according to benign biopsy rates. A total of 34 biopsies were performed in the entire cohort with 25 biopsies based on MRI interpretations. The false-positive rate based on a benign biopsy for the entire group was 5.9% (22/371) a rate of 6.1% (10/165) for the stacked group and 5.8% (12/206) for the alternating group. There was not a significant difference between false-positive rates based on having a benign biopsy for the stacked compared to alternating group (p = 0.92).

Four breast cancers were detected in 137 women included in this cohort. Three were identified by MRI (1 on a stacked schedule and 2 on an alternating schedule). There was 1 interval cancer in this cohort. Table 4 demonstrates the characteristics of the cancers identified. No cancers were identified in women within 1 year of having a BI-RADS 3 category MRI.

**Table 4:** **Characteristics of breast cancers diagnosed in cohort**

**Preceding MRI type**	**MRI BI-RADS category**	**MRI cycle #**	**Biopsy result**	**Breast cancer risk factor**
**Stacked**	4	1	0.8 cm MD IDC, LN -, ER/PR+, HER 2-	Hx of LCIS
**Alternating**	5	3	0.7 cm MD ILC, LN-, ER/PR +, HER 2-	BRCA 2 carrier
**Alternating**	0	5	0.6 cm MD ILC, LN-, ER+, PR+	BRCA 2 carrier
**Interval cancer**		5	2.4 cm PD IDC, LN+, ER+, PR-, HER 2 -	BRCA 1 carrier

Abbreviations: MD = moderately differentiated, PD = poorly differentiated, IDC = infiltrating ductal carcinoma, ILC = infiltrating lobular carcinoma, LN = lymph node, ER = estrogen receptor, PR = progesterone receptor.

## Discussion

In this retrospective evaluation of a prospectively gathered cohort we have shown similar rates of false positive studies regardless of the screening schedule or definition of a positive study. To our knowledge, this is one of the first studies to compare the two styles of screening in a prospectively gathered cohort and one of the largest series to examine the alternating schedule of mammogram and screening breast MRI. Le-Petross et al. reported their institutional practice of alternating studies and found a sensitivity of 92% and a specificity of 79%; similar to published series with studies performed annually (Brekelmans et al. 2001) (Kriege et al. 2001) (Tilanus-Linthorst et al. 2000) (Kriege et al. 2004) (Weinstein et al. 2009) (Klijn 2010) (Kuhl et al. 2005) (Warner et al. 2004) (Leach et al. 2005; Le-Petross et al. 2011). While their study suggests that imaging performed on an alternating schedule has similar sensitivity and specificity to imaging performed on a stacked schedule, they did not compare the two strategies. One additional study suggests that alternating imagining is a more cost efficient strategy, especially for BRCA1 carriers, largely related to the high incidence of cancer (Cott Chubiz et al. 2013) (Lowry et al. 2012).

In our cohort false-positive rates were influenced by both the type of examination and the definition of a positive study. We observed a higher overall false positive rate for baseline screening MRI compared to subsequent examination (24.8% vs 9.5%) as highlighted in Table 3. Higher false-positive rates for baseline compared to subsequent mammograms have been reported (Frankel et al. 1995) (Burnside et al. 2002) (Callaway et al. 1997). Only one study has examined this issue for screening MRI (Abramovici & Mainiero 2011). Using data from the Dutch MRI Screening (MRISC) study the false positive rate for the baseline screening breast MRI was 14% which decreased to 8.2% with subsequent rounds of screening. We did not find a significant difference between false-positive rates for baseline or subsequent examinations based on schedule.

We did observe a significant difference in false-positive rates depending on the definition of a positive study (BI-RADS 4, 5, 0 vs BI-RADS 3, 4, 5, 0 or biopsy). We found the highest false-positive rate when considering BI-RADS 3, 4, 5, 0 as a positive study (13.7%), with much lower rates of false-positive studies for BI-RADS 4, 5, 0 (7.8%) and for benign biopsy (5.9%). The higher rate when calling BI-RADS 3, 4, 5, 0 positive can be explained by BI-RADS 3 interpretations. In our cohort there were no cancers identified within one year of any BI-RADS 3 MRI. Others have shown similarly high rates of false positive screening breast MRI when including BI-RADS 3 interpretations as positive, with a range between 6.3 – 27% in published studies (Bahrs et al. 2014) (Eby et al. 2009) (Liberman et al. 2003) (Weinstein et al. 2010) (Eby et al. 2007) (Hauth et al. 2010) (Kuhl et al. 2005) (Rosen et al. 2002) (Barr et al. 2013). The high rate of BI-RADS 3 lesions and low likelihood of malignancy associated with a BI-RADS 3 lesions for any breast screening modality (MRI, mammogram or ultrasound) has prompted some to suggest that short-interval follow-up may not be needed for this group (Bahrs et al. 2014) (Barr et al. 2013) (Varas et al. 2002) (Vizcaino et al. 2001).

The number of cancers identified in our population is quite small, likely due to the broader definition of “high risk” and short follow-up time. Three of the four cancers were identified by screening and these were all lymph node negative and less than 1 cm (Table 4). There was one interval cancer in a BRCA1 carrier which was lymph node positive and >2 cm. This low rate of interval cancers is similar to other studies combining screening breast MRI with annual mammography (Kriege et al. 2004) (Warner et al. 2004) (Leach et al. 2005) (Kuhl et al. 2005) (Sardanelli et al. 2011). Similar to Warner et al. we have shown that screening with MRI is associated with identification of lower risk cancers (Warner et al. 2011) (Gareth et al. 2014).

This study has weaknesses that must be considered. Women in this study were not randomized to a screening schedule and there is likely some selection bias to the choices made for screening schedule. The cohort is heterogeneous with respect to breast cancer risk; including women with BRCA mutations, women with strong family histories and women who had a biopsy showing atypia. Despite these weaknesses this study has several strengths. This is the largest cohort to date reporting screening on different schedules. Le-Petross et al. studied 73 women with BRCA1 and BRCA2 mutations who were getting every 6 month (alternating) screening only (Le-Petross et al. 2011). In our cohort of high risk women the decision of which schedule to adhere to (alternating or stacked) was made by the provider and patient, making it similar to a real –world experience. The majority of women (71%) kept to the chosen schedule through multiple rounds of screening; despite the fact that this population is young and largely (79%) premenopausal.

In conclusion, we have shown that the false-positive rates are similar for studies performed on either a stacked or alternating schedules regardless of the definition of a false-positive study. This finding and information from prior studies (Le-Petross et al. 2011) showing similar sensitivity for alternating schedules, suggests that high risk women and their providers may choose the schedule they prefer when screening with annual MRI and mammogram. This is important as there is not likely to be a randomized clinical trial comparing the two schedules given the high cost and large number of women needed for such a study.
